# Provider adherence to first antenatal care guidelines and risk of pregnancy complications in public sector facilities: a Ghanaian cohort study

**DOI:** 10.1186/s12884-016-1167-6

**Published:** 2016-11-24

**Authors:** Mary Amoakoh-Coleman, Kerstin Klipstein-Grobusch, Irene Akua Agyepong, Gbenga A. Kayode, Diederick E. Grobbee, Evelyn K. Ansah

**Affiliations:** 1 Postdoctoral Unit, Noguchi Memorial Institute for Medical Research, University of Ghana, Legon, Accra Ghana; 2 Julius Global Health, Julius Center for Health Sciences and Primary Care, University Medical Centre Utrecht, Utrecht, The Netherlands; 3 Department of Epidemiology and Disease Control, School of Public Health, University of Ghana, Legon, Ghana; 4 Division of Epidemiology & Biostatistics, School of Public Health, Faculty of Health Sciences, University of the Witwatersrand, Johannesburg, South Africa; 5 Department of Health Policy, Planning and Management, School of Public Health, University of Ghana, Legon, Ghana; 6 Research and Development Division, Ghana Health Service, Accra, Ghana

**Keywords:** Adherence, Antenatal care, First antenatal visit, Maternal, Neonatal, Outcomes

## Abstract

**Background:**

Guideline utilization aims at improvement in quality of care and better health outcomes. The objective of the current study was to determine the effect of provider complete adherence to the first antenatal care guidelines on the risk of maternal and neonatal complications in a low resource setting.

**Methods:**

Women delivering in 11 health facilities in the Greater Accra region of Ghana were recruited into a cohort study. Their first antenatal visit records were reviewed to assess providers’ adherence to the guidelines, using a thirteen-point checklist. Information on their socio-demographic characteristics and previous pregnancy history was collected. Participants were followed up for 6 weeks post-partum to complete data collection on outcomes. The incidence of maternal and neonatal complications was estimated. The effects of complete adherence on risk of maternal and neonatal complications were estimated and expressed as relative risks (RRs) with their 95% confidence intervals (CI) adjusted for a potential clustering effect of health facilities.

**Results:**

Overall, 926 women were followed up to 6 weeks post-partum. Mean age (SD) of participants was 28.2 (5.4) years. Complete adherence to guidelines pertained to the care of 48.5% of women. Incidence of preterm deliveries, low birth weight, stillbirths and neonatal mortality were 5.3, 6.1, 0.4 and 1.4% respectively. Complete adherence to the guidelines decreased risk of any neonatal complication [0.72 (0.65–0.93); *p = 0.01*] and delivery complication [0.66 (0.44–0.99), *p* = 0.04].

**Conclusion:**

Complete provider adherence to antenatal care guidelines at first antenatal visit influences delivery and neonatal outcomes. While there is the need to explore and understand explanatory mechanisms for these observations, programs that promote complete adherence to guidelines will improve the pregnancy outcomes.

**Electronic supplementary material:**

The online version of this article (doi:10.1186/s12884-016-1167-6) contains supplementary material, which is available to authorized users.

## Background

With respect to Millennium Development Goal 5 (MDG 5), substantial progress has been achieved in almost all regions, except in sub-Saharan Africa, which could not reach the set target of reducing maternal mortality by 75% by 2015 [1]. Given Ghana’s progress so far, maternal mortality was estimated to be reduced to only 340 per 100,000 by the end of 2015 instead of the MDG target of 185 per 100,000 [2–4].

Efforts made to help Ghana meet the MDG included adoption of the Safe Motherhood Initiative (SMI) launched globally in 1987, together with other policy introductions such as free antenatal care for all pregnant women in 1998 and exempting all users from delivery fees in health facilities in 2003 [5].

One of the public health interventions aimed at alleviating complications of pregnancy and childbirth is antenatal care [6, 7]. The first antenatal care visit offers the opportunity to identify women who are likely to have unfavourable pregnancy outcomes [8]. Guidelines for antenatal care exist to support the health care provider to maximize this potential.

Adherence to clinical guidelines is important in ensuring uniformity of clinical care, as well as maintaining quality service provision to patients based on their specific needs [9]. This has also been shown to lead to improved clinical outcomes [10–12]. Studies measuring the effects of guidelines on quality of care have shown significant improvements in the process of care [13–16]. Other studies focusing on effects on patient health outcomes [17–21] have demonstrated improved outcomes with increased adherence [17, 18, 22–24]. Most of these studies however have been conducted in advanced resource settings, and focused on non-obstetric medical and surgical conditions. In Ghana, studies that have evaluated adherence to guidelines are limited [25–27] with few focusing on antenatal care guidelines.

Adherence to guidelines has been shown to vary considerably in many settings and often remain low [8, 26–28], translating into poor health outcomes. As part of the SMI implementation in Ghana, various tools including protocols and guidelines such as the National Safe Motherhood Service Protocol (SMP) were developed to assist health workers in caring for pregnant women.

Our objective was to determine the effect of provider complete adherence to the first antenatal care guidelines on the risk of maternal and neonatal complications.

## Methods

### Study design

A cohort study with both retrospective and prospective arms was conducted.

### Study setting

The study was conducted in the Greater Accra Region (GAR) of Ghana. The region is about 90% urban, and is served by both public and private facilities. About 97% of pregnant women here attend antenatal clinic at least once during their pregnancy and skilled attendance at delivery is around 84%. About 62% of all health facility deliveries in the region take place in the public sector. The public sector comprises of one teaching hospital, a regional hospital and nine district and sub-metropolitan hospitals. There are ten polyclinics, 31 health centres, some community clinics and three Community Health Planning and Services (CHPS) compounds. The polyclinics are primary health care facilities like the health centres, usually sited in urban and populous areas and provide both general and specialist services. They also have more than one doctor unlike the health center which usually has one doctor or in some cases manned by a medical assistant. CHPS compounds operate at the community level, with midwives or community health nurses, either through home visits or clients going to the compounds. There are several private hospitals operating in the region. All these health facilities offer antenatal and delivery services, though the smaller facilities refer complications to the bigger health facilities. The National Health Insurance Scheme (NHIS) is operational in all the public as well as in most of the private facilities.

### Selection of districts and facilities

We randomly selected participants from the different levels of public health care (variable “type of facility”) across the Greater Accra region. All districts and sub-metropolises in the region were grouped into those with a district or sub-metropolitan hospital (eight in number) and those without hospitals (12 in total) and then five districts were randomly selected by balloting with replacement from each group. By this process, the names of the districts in each group were written on a piece of paper, folded and put in a box. After shuffling by an independent person, five districts were picked one at a time, replacing the picked district in the box each time before picking the next to ensure equal chance of selection for all. The district hospitals in the selected districts with hospitals were included in our study. In those districts without hospitals all the primary level care facilities offering both antenatal and delivery services were included for random sampling and one was selected from each district.” We also included the regional hospital which is in a sub-metropolis different from the selected districts/metropolises. Thus in all, 11 health facilities (one regional and five district/sub-metropolitan hospitals, four polyclinics and a health center) were randomly selected from 11 districts/sub-metropolises for the study.

### Sample size and recruitment of women

The sample size for the cohort study was based on a prevalence of pregnancy complications of 6% [29] using Open Epi calculator for estimation. In the absence of any documented evidence in the setting, we assumed that the complication rate will be twice as high amongst the unexposed group (incomplete adherence). To detect a two-sided significance difference at 95% confidence interval at a power of 80%, and a one-to-one ratio of exposure to non-exposure, a sample of 372 women was required per exposure group. The minimum total sample size therefore required for both arms was 744 women.

Participants were recruited at delivery and once they met the inclusion criteria, informed consent was obtained. The inclusion criteria included the following: participant 18 years or older; participant had at least one antenatal clinic visit in a health facility during the current pregnancy; participant had first antenatal clinic visit at gestational age less than or equal to 5 months and participant had first antenatal clinic visit at the facility of delivery or in one of the sampled facilities for this study.

From each of the eleven (11) facilities sampled for the study, we recruited a minimum of 68 women, a minimum of 34 women exposed to complete provider adherence and a minimum of 34 unexposed women. We continued recruitment irrespective of adherence status until we had at least 34 women in either group.

### Data collection processes and tools

Every woman delivering at the facility on any day and who met the inclusion criteria, and provided informed consent for participation, was enrolled into the study.

At recruitment, a record review of their first antenatal clinic visit from the maternal health record book was retrospectively carried out using a checklist. Data on socio-demographic characteristics, potential confounders such as timing of first antenatal visit, number of antenatal clinic attendance and previous pregnancy history, as well as the 13 variables on guideline requirements were collected. Records of subsequent antenatal clinic visits were also reviewed for any complications developed and identified during the pregnancy. Finally, data on delivery outcomes was collected from both the maternal health record book and delivery register and notes. Participants’ telephone numbers were linked to their study identification numbers (IDs) for follow up.

In the prospective arm, all women and their neonates were followed up till 6 weeks postpartum to complete data collection on outcomes. Follow up was at the health facility during post-natal care visit at 6 weeks postpartum and also by phone. Participants received a phone call 3 weeks post-partum, and at 6 weeks, they were followed up at the postnatal clinic. If they were not available there, they received another phone call. Those who could not be reached by any of these means were treated as lost to follow up. A facility audit was conducted to assess facility factors such as the availability of personnel, services, infrastructure, logistics and supplies that are needed to support adherence to the guidelines at facility level.

The assumption was made that any information on history, examination, laboratory examination and treatment available is what was recorded in the maternal health record book. Information on any service not recorded, was deemed not to have been delivered [30].

### Variables

The outcome variables studied were all maternal and neonatal complications during antenatal, delivery and post-partum periods. Maternal complications were i. any antenatal complication (defined as having at least one of the following: anemia in pregnancy, pregnancy induced hypertension/pre-eclampsia/eclampsia, malaria in pregnancy and antepartum vaginal bleeding), ii. Any antenatal complication excluding anemia in pregnancy,, iii. Caesarean section, iv. Delivery complication (defined as having at least one of the following: pregnancy induced hypertension/pre-eclampsia/eclampsia, ruptured uterus, obstructed labour, vaginal tears and perineal tears), v. post-delivery complication (defined as having at least one of the following: postpartum haemorrhage, anemia, malaria and sepsis), vi. Maternal mortality, vii. Any maternal complication (which is defined as having at least one of the listed maternal complications). Neonatal complications were preterm, post-maturity, low birth weight, neonatal jaundice, asphyxia, still birth and neonatal mortality.

The determinants were type of facility, client’s socio-demographics (age, educational level, marital status and employment status), and client’s prenatal factors (parity, trimester at first antenatal care visit, previous pregnancy history and number of times antenatal clinic was attended during pregnancy). Provider adherence levels were defined as complete or incomplete.

#### Measuring adherence

Several studies have measured adherence using a scoring system, based on available guidelines or protocol requirements [21, 31–33]. In this study, a thirteen-point checklist was used to score provider adherence to first antenatal visit guidelines. Adherence to first antenatal guidelines was used as a proxy for adherence to guidelines in general. The questions on the checklist were based on the requirements for first antenatal visit as per the SMP for Ghana, which is also consistent with the national treatment guidelines for first antenatal visit. They related to if: Age recorded; Parity recorded; Gestational age at booking recorded; Last pregnancy history if applicable recorded**;** Medical, surgical or family history recorded; Weight recorded; Blood pressure recorded; Abdomen examined; Haemoglobin test done; Urine test done; Iron supplement given; Tetanus injection given or status recorded; Intermittent preventive treatment of malaria (IPTp) given. Eleven of the variables on the checklist were referred to as “mandatory variables” while two of them were referred to as “optional” variables. The “optional variables may not be due at the first antenatal visit, depending on the woman’s gestational age and therefore do not influence the adherence categorization. These are the “last pregnancy history if applicable” and “Intermittent Presumptive Treatment in pregnancy (IPTp) given if woman is due”. IPTp is indicated for women in the second and third trimesters only. We included the “optional” variables in order to describe how they contribute to antenatal care quality. Every record reviewed was assessed to see how many of the 13 variables were actually adhered to by the provider at the first antenatal visit.

Each variable adhered to, scored a point of 1 while non-adherence scored 0. A total score of 11–13 (including a score of 1 to all the 11 mandatory variables) was classified as complete adherence to guidelines. Non-adherence to any of the mandatory variables was classified as incomplete adherence. The adherence checklist, scoring criteria and entire methodology for the study has been published elsewhere [34] and is provided as Additional file 1.

### Data analysis

Descriptive analysis of participants’ socio-demographic information and previous pregnancy history was conducted by use of frequencies and chi-square analysis. Adherence to guidelines was computed by calculating the proportion of women whose first antenatal clinic visits met the criteria for complete adherence. Possible associations of adherence status with some patient characteristics were assessed using chi-square. Incidences of maternal and neonatal complications were estimated in percentages. Determinants of maternal and neonatal complications, as well as the effect of complete adherence on pregnancy outcomes were estimated and expressed as relative risks (RRs) with their 95% confidence intervals (CI). To correct for potential clustering effect due to our sampling strategy, we used generalized linear equation estimation in calculating RRs. We also adjusted for variables such as trimester of first ANC visit, maternal age, parity, previous pregnancy complication and any antenatal complication where appropriate. Significance was determined at *p-value <0.05*. Data analysis was carried out using IBM SPSS Statistics for Windows, Version 20.0. Armonk, NY: IBM Corp.

## Results

**Fig. 1:**
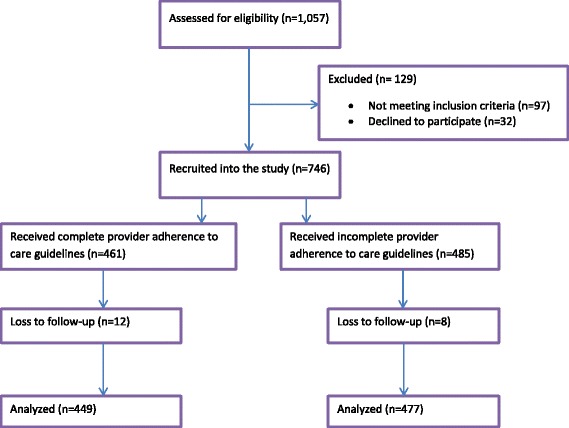
Flow diagram for participants’ in the study. A description of participants’ recruitment, follow-up and analysis in the study

A total of 946 women were recruited and 926 of them were followed up to 6 weeks post-partum from December 2013 to May 2014. We recruited more women than the minimum required because the minimum number of women per each group had to be satisfied. In places where more women with complete adherence were included, we continued recruitment until we reached the minimum of 34 for the incomplete adherence group, and vice versa. Figure 1 is a flow chart of participants’ recruitment and follow-up in the study.

Fifty-six percent of the women were seen at six hospitals, while 36.0% were seen at four polyclinics and 8.0% at a health center.

### Adherence to first antenatal care guidelines

Overall, complete adherence to guidelines pertained to the care of only 48.5% [95% CI (45.3–51.7%)] of all participants in our sample, during their first antenatal clinic visit. The range was 33.8 to 61.7% amongst the facilities sampled. Complete adherence to guidelines was higher amongst women seen at the polyclinics [52.9% (47.5–58.2%)] than those seen in hospitals [47.8% (43.5–52.1%)] and the health center [34.2% (22.8–44.8%)].

### Participants’ baseline characteristics

The mean age (SD) of participants was 28.2 (5.4) years. About 85% of them were aged 20–35 years. Less than half of participants had secondary education, with 11.8 and 19.3% having no education and primary education respectively. Married women made up 72.6% of all participants and most of the women (84.9%) were employed, 68.1% had their first antenatal visit during the second trimester and 78.1% attended at antenatal clinic at least four times during the pregnancy. Only 7.7% of the women had a history of previous pregnancy complication and 45.6% of the women were anemic at their first antenatal visit. Details of the baseline characteristics for the two groups of participants are given in Table 1.

**Table 1:** Baseline characteristics of study participants and association of these characteristics with levels of provider adherence to first antenatal care (ANC) guidelines

Variable	Frequency (%)	% Incomplete adherence	% Complete adherence	Cluster adjusted
	*N* = 926	*N* = 477	*N* = 449	*p*-value
Total sample		51.5	48.5	
Facility type				<0.01
Hospital	519 (56.0)	52.2	47.8	
Polyclinic	333 (36.0)	47.1	52.9	
Health center	74 (8.0)	65.8	34.2	
Age category				0.01
<20 years	52 (5.6)	73.1	26.9	
20–35 years	790 (85.5)	50.5	49.5	
> =35 years	82 (8.9)	47.6	52.4	
Parity				0.01
0	258 (27.9)	76.7	22.7	
1–2	475 (51.3)	40.8	59.2	
3–4	162 (17.5)	43.2	56.8	
>4	31 (3.3)	48.4	51.6	
Education				0.87
None	109 (11.8)	47.7	52.3	
Primary	179 (19.3)	47.5	52.5	
Secondary	423 (45.7)	51.8	48.2	
Tertiary	163 (17.7)	57.1	42.9	
Other	46 (5.0)	47.8	52.2	
Trimester at first ANC				0.05
First	282 (30.5)	57.8	42.2	
Second	631 (68.1)	48.0	52.0	
Number of times ANC attended				<0.01
1	44 (4.8)	79.5	20.5	
2–3	137 (14.8)	48.9	50.7	
> = 4	723 (78.1)	50.1	49.9	
Marital status				0 01
Single	100 (10.8)	64.0	20.5	
Married	672 (72.6)	48.5	51.5	
Formerly married	12 (1.3)	58.3	41.7	
Living together	122 (13.2)	54.9	45.1	
Employment				0.01
No	141 (15.2)	66.0	34.0	
Yes	777 (84.9)	48.8	51.2	
Previous pregnancy history				0.04
No complication	853 (92.1)	51.2	48.8	
Mean age (SD)	28.15 (5.4)	27.25 (5.6)	29.11 (5.0)	<0.01

### Risk of maternal and neonatal complications

Table 2 shows the incidence of maternal and neonatal complications. Overall, 68.1% of the women developed antenatal complications. As many as 59.4% of the women had anemia during the pregnancy and 15.5% developed anemia after the first antenatal visit. Incidence of pregnancy-induced hypertension and its complications and post-partum hemorrhage were 5.3 and 2.1% respectively. Risk for any pregnancy, delivery or postpartum complication was 68.6%. There was no maternal mortality amongst the study participants. Incidence of preterm deliveries, low birth weight babies, stillbirths and neonatal mortality were 5.3, 6.1, 0.4 and 1.4% respectively. There were differences in risks of most of the complications amongst the two adherence groups, but these were not statistically significant.

**Table 2:** Risk of antenatal, delivery and postpartum complications amongst cohort and comparison of risks between the 2 adherence groups

Complication	Incidence (%)	Complete adherence	Incomplete adherence	Cluster adjusted
	*N* = 926	*N* = 449	*N* = 477	*p*-value
Maternal				
Malaria in pregnancy	113 (12.2)	56 (12.5)	57 (11.9)	0.81
Antepartum vaginal bleeding	10 (1.1)	3 (0.7)	7 (1.5)	0.24
PIH/pre-eclampsia/eclampsia	49 (5.3)	23 (5.1)	26 (5.5)	0.82
Anemia in pregnancy	550 (59.4)	269 (59.9)	281 (58.9)	0.12
Anemia developed after first antenatal visit	144 (15.5)	67 (14.9)	77 (16.1)	0.36
Any antenatal complication	631 (68.1)	308 (68.6)	328 (68.7)	0.88
Any antenatal complication (non-anemia)	207 (22.4)	99 (22.0)	108 (22.6)	0.83
Delivery by Caesarean section	132 (14.3)	65 (14.5)	67 (14.0)	0.84
Post-partum hemorrhage	19 (2.1)	6 (1.3)	13 (2.7)	0.14
Delivery/post-delivery complication	41 (4.4)	17 (3.8)	24 (5.0)	0.82
Any pregnancy related complication	635 (68.6)	310 (69.0)	325 (68.1)	0.87
Any pregnancy related complication (non-anemia)	229 (24.4)	108 (24.1)	121 (25.4)	0.56
Maternal mortality	0 (0.0)	0 (0.0)	0 (0.0)	
Neonatal				
Preterm	49 (5.3)	23 (5.1)	26 (5.5)	0.31
Post-maturity	88 (9.5)	36 (8.0)	52 (10.9)	0.31
Low birth weight	57 (6.1)	19 (4.2)	36 (7.5)	0.21
Neonatal jaundice	58 (6.3)	29 (6.5)	29 (6.1)	0.21
Asphyxia/difficulty in breathing	39 (4.2)	15 (3.3)	34 (7.1)	0.21
Stillbirths	4 (0.4)	2 (0.4)	2 (0.4)	0.21
Any neonatal complication	146 (15.8)	60 (13.4)	86 (18.0)	0.05
Neonatal mortality (All cause)	13 (1.4)	6 (1.3)	7 (1.5)	0.87

In the univariable analysis, provider adherence significantly influenced both neonatal and any maternal complication (antenatal, delivery or post-partum) (Table 3). Neonatal complications were reduced by almost 30.0% amongst women whose first ANC care was standard as per the guidelines while delivery related complication for such women was reduced by about 40.0%. Controlling for potential confounders in a multivariable analysis only slightly altered risk estimates for neonatal [0.72 (0.56–0.93), *p* = 0.01], and delivery related complications [0.66 (0.33–0.99), *p* = 0.04], (Table 4).

**Table 3:** Univariable association between independent variables and antenatal, delivery and post-partum complications among study participants

Independent factor	Antenatal complication RR (95% CI)	*p-value*	Delivery/postpartum complication RR (95% CI)	*p-value*	Any maternal complication RR (95% CI)	*p-value*	Neonatal complication RR (95% CI)	*p-value*
Complete adherence	*1.02 (0.70–.50)*	*0.90*	0.73 (0.48–1.11)	0.14	0.64 (0.43–0.94)	0.03	0.73 (0.55–0.96)	*0.03*
Employment (Yes)	*0.72 (0.4–1.10)*	*0.13*	2.17 (1.17–4.01)	*0.01*	1.19 (0.63–2.95)	*0.58*	0.89 (0.60–1.32)	*0.56*
Age categories								
<20 years	Ɨ		Ɨ		Ɨ		Ɨ	
20–35years	1.20 (0.76–1.91)	*0.44*	0.60 (0.26–1.40)	*0.24*	0.42 (0.28–0.64)	*<0.01*	0.60 (0.31–1.34)	*0.12*
>35 years	0.74 (0.39–1.41)	*0.36*	1.72 (0.64–4.63)	*0.29*	1.04 (0.68–1.58)	*0.85*	0.37 (0.12–1.13)	*0.08*
Parity								
0	Ɨ		Ɨ		Ɨ		Ɨ	
1–2	0.91 (0.71–1.71)	*0.48*	1.56 (0.51–4.73)	*0.43*			0.68 (0.48–0.95)	*0.03*
3–4	1.49 (0.97–2.31)	*0.07*	2.80 (1.47–5.35)	*<0.01*			0.68 (0.43–1.07)	*0.10*
>4	0.72 (0.31–1.71)	*0.46*	1.11 (0.38–3.20)	*0.85*			0.59 (0.25–6.41)	*0.24*
Trimester of first ANC attendance								
First	Ɨ		Ɨ		Ɨ		Ɨ	
Second	1.10 (0.72–1.68)	*0.65*	0.87 (0.35*–*2.17)	*0.77*	0.82 (0.41*–*1.69)	*0.57*	0.86 (0.53*–*1.38)	*0.53*
Number of times ANC attended (> = 4)	0.73 (0.28*–*1.84)	*0.50*	0.46 (0.11*–*1.98)	*0.30*	0.63 (0.17*–*2.34)	*0.49*	1.01 (0.19*–*1.77)	*0.33*
Previous delivery complication (Yes)	1.11 (0.64*–*1.94)	*0.71*	1.13 (0.50*–*2.59)	*0.76*	1.11 (0.31*–*3.91)	*0.88*	1.24 (1.81*–*1.92)	*0.32*
Any antenatal complication	NA		0.87 (0.54*–*1.37)	*0.54*	NA		1.55 (1.10*–*2.19)	*0.01*
Antenatal complication (non-anemia)	NA		1.92 (1.09*–*3.38)	*0.02*	NA		2.67 (1.86*–*3.83)	*<0.01*

*ANC* denotes antenatal clinic*, CI* denotes confidence interval*, NA* denotes not applicable, *RR* denotes relative risk

**Table 4:** Effect of complete adherence on risk of pregnancy complications

Complication	Crude RR 95% Confidence interval	*p-value*	^a^Adjusted RR 95% Confidence interval	*p-value*
Neonatal	0.73 (0.55*–*0.96)	0.03	0.72^c^ (0.56*–*0.93)	*0.01*
Any maternal	1.01 (0.67*–*1.48)	0.97	1.00^b^ (0.67*–*1.48)	*0.99*
Antenatal	1.02 (0.70*–*1.50)	0.90	1.02^b^ (0.69*–*1.52)	*0.92*
Antenatal (non-anemia)	0.96 (0.75*–*1.23)	0.73	0.90^b^ (0.66*–*1.22)	*0.45*
PIH/pre-eclampsia/eclampsia	0.92 (0.62*–*1.37)	0.69	0.95^b^ (0.57*–*1.58)	*0.84*
Antepartum vaginal bleeding	0.45 (0.17*–*1.21)	0.11	0.48^b^ (0.16*–*1.43)	*0.19*
Malaria in pregnancy	1.05 (0.70*–*1.58)	0.80	1.02^b^ (0.68*–*1.53)	*0.92*
Delivery	0.64 (0.43*–*0.94)	0.03	0.66^b^ (0.44*–*0.99)	*0.04*
Post-partum	0.71 (0.28*–*1.77)	0.46	0.72^b^ (0.33*–*1.59)	*0.42*

*PIH* denotes pregnancy induced hypertension, *RR* denotes relative risk

^a^All RR adjusted for possible clustering effect due to sampling strategy

^b^Estimate adjusted for marital status, employment, number of times ANC was attended, trimester of first antenatal visit, maternal age, parity and previous pregnancy complication

^c^Estimate adjusted for marital status, employment, number of times ANC was attended trimester of first antenatal visit, maternal age, parity, previous pregnancy complication and any antenatal complication

## Discussion

### Main findings

Key findings of this study are that provider adherence to first antenatal care guidelines is low (48.5%), most women register for first antenatal care in the second trimester and complete adherence to first antenatal guidelines reduced the risk of delivery and any neonatal complication.

### Strengths and limitations

Adherence to first antenatal care guidelines was retrospectively scored from participants’ records. It is therefore not possible that provider practice changed because of the study, thus reflecting everyday provider practice of adherence to guidelines. Loss of follow-up was minimal at 2.1%. We however recognize some limitations for our study. The fact that we recorded zero maternal deaths in our study is not reflective of the situation in Greater Accra, and might purely be due to the fact most of our participants are those who survived pregnancy and delivery. Also, since only women receiving care in public facilities were included in the study, generalizability of the results to service delivery in private practice is limited.

### Interpretation

Antenatal care as well as skilled attendance at delivery (which all participants in our study had) have been shown to improve maternal and neonatal outcomes [35]. Provider adherence to first antenatal care guidelines was observed to be low in our study in contrast to what other studies [36–38] conducted in advanced settings found. However, one earlier study found limited use of maternal health guidelines in Ghana, Burkina Faso and Tanzania [39]. Previous studies from Ghana identified gaps in quality of care of maternal, new-born and child health, including antenatal care [40, 41], which may be explained by low adherence to guidelines and subsequent suboptimal quality of care provided to pregnant women.

The current study demonstrates the significant role of quality of care in translating access to antenatal care to good outcomes. Complete adherence to first antenatal guidelines resulted in reduced risk of delivery and neonatal complications. Guidelines can only lead to improved or quality care if they are translated into daily provider practice [42]. As they continue to provide service, clinicians may not even be aware of their lack of adherence to available guidelines [43]. It has been established that active steps, such as reminders, are necessary to translate clinical practice guidelines into daily practice [9, 42, 44]. The process of engagement of and support for providers of maternal and neonatal care with regards to utilization of clinical guidelines can be enhanced through a mechanism of regular monitoring and providing feedback to them as has been done in other contexts [45]. Programs to improve adherence to guidelines that are fashioned around a continuous educational framework for providers are likely to transform provider practice [46] and will subsequently improve the outcome of neonatal service delivery.

Provider adherence to guidelines however did not significantly influence the risk of any maternal complications, although it specifically reduced the risk of delivery related complications. Perhaps other mechanisms better explain maternal outcomes apart from adherence. It must also be recognized that perhaps the frequent antenatal clinic attendance allows providers to make up for what they missed out during the first antenatal visit. Basic, skilled and ultimately safe intrapartum care is noted to be important in addressing maternal and neonatal morbidity and mortality [2, 47], and any intervention of the process that results in better outcomes should be encouraged. Evidence based practice intrapartum is essential as one systematic review has shown [48]. Thus mechanisms to enhance provider adherence to both ANC and delivery guidelines should be instituted at all levels of care.

Although poor quality of care decreases utilization of services [41], we found that most women in our study attended antenatal clinics at least four times during their pregnancy despite low provider adherence to guidelines during their first visit. It is uncertain how much of guideline requirement is known by clients, but one study has described high satisfaction for antenatal care services amongst attendants in Ghana [49]. Frequent antenatal attendance provides enough contact between the provider and the pregnant woman to identify and address any challenges of the pregnancy [50]. It is also important that during each of these contacts quality of care is assured.

We also find that women are reporting late for their first antenatal visit. Most women reported during the second trimester, as previously noted in studies both in Ghana and other low and middle-income countries [6, 51–55]. This is a source of concern as pregnancy complications may be recognized too late and the opportunity for timely interventions missed.

About 70% of the study participants had at least one form of complication during the antenatal, delivery or post-partum period. The majority of these complications occurred during antenatal period and anemia in pregnancy was high on the list. This is worrying in a setting where hemorrhage is a common cause of maternal deaths [56], since anemia will affect the woman’s ability to adequately compensate physiologically in case of bleeding during delivery. We are not sure what proportion of this risk of anemia is due to physiological hemodilution in pregnancy [57], but malaria is a common cause of anemia in pregnancy in our setting [57–59], the incidence being 12.2% amongst our study participants. Both malaria in pregnancy and anemia potentially increase the risk of low birth weight and prematurity [58]. Compared to other studies, risk of hemorrhage was low (2.1%) amongst our study participants compared to what other studies have found [56, 60]. The relatively high rate of caesarean section in our study may partly be due to the fact many of our facilities were hospitals which receive referrals from lower level facilities. However, this was within the range of globally the acceptable rates of up to 15% [29, 61].

Previous pregnancy history was found to be an important determinant of maternal outcome as shown in our study and other studies [62, 63] and so providers can use this information in their clinical decision making during health care provision to pregnant women.

In the year 2013, the Greater Accra region recorded as many as 201 institutional maternal deaths [64]. For the 6 months of data collection within 11 facilities in the region no maternal death was recorded amongst our participants post-delivery. Maternal deaths are usually a result of direct causes like hemorrhage, pre-eclampsia and eclampsia and sepsis as well as indirect causes like anemia [65]. These occur mostly during delivery or within 24 h afterwards [65, 66]. Although no maternal deaths occurred amongst our participants during the 6 weeks postpartum, it is possible that we missed some deaths that may have occurred before or during delivery since we recruited women who had already survived delivery or that our sample was not large enough to detect such an outcome in the postpartum period.

## Conclusion

Complete provider adherence to first antenatal care guidelines was shown to influence neonatal outcomes but showed no effect on maternal outcomes. There is the need to explore and understand the possibility of explanatory mechanisms for these observations. Also, since adherence to first antenatal visit guidelines were used as a proxy for provider adherence to guidelines in general, we believe programs that promote complete adherence to guidelines will improve the outcome of neonatal service delivery. Early antenatal care should also be encouraged amongst the population.

## Additional file

Additional file 1: Table showing the checklist for scoring provider adherence to antenatal guidelines. (DOCX 20 kb)

## Abbreviations

ANC: Antenatal clinic
CHPS: Community based health and planning services
CI: Confidence interval
ERC: Ethics review committee
GAR: Greater Accra Region
GHS: Ghana health service
IPTp: Intermittent presumptive treatment in pregnancy
MDG: Millennium development goal
NHIS: National Health Insurance Scheme
SD: Standard deviation
SMI: Safe motherhood initiative
