# Supplementary material for: Provider adherence to first antenatal care guidelines and risk of pregnancy complications in public sector facilities: a Ghanaian cohort study
Source: BMC Pregnancy Childbirth. 2016 Nov 24;16:369. doi: 10.1186/s12884-016-1167-6 (PMC5121950; doi:10.1186/s12884-016-1167-6)
Supplement: Additional file 1: — Table showing the checklist for scoring provider adherence to antenatal guidelines. (DOCX 20 kb) [file 12884_2016_1167_MOESM1_ESM.docx]

**Supplement 1: Variables on adherence checklist for first ANC guidelines and scoring criteria**

| Variable checked  **** Optional** variables  Others are **Mandatory** | Yes (1) | No (0) |
| --- | --- | --- |
| 1. Age recorded |  |  |
| 1. Parity recorded |  |  |
| 1. Gestational age at booking recorded |  |  |
| 1. Last pregnancy history if applicable recorded****** |  |  |
| 1. Medical, surgical or family history recorded |  |  |
| 1. Weight recorded |  |  |
| 1. Blood pressure recorded |  |  |
| 1. Abdomen examined |  |  |
| 1. Hemoglobin test done |  |  |
| 1. Urine test done |  |  |
| 1. Iron supplement given |  |  |
| 1. Tetanus injection given or status recorded |  |  |
| 1. Intermittent preventive treatment of malaria (IPTp) given if woman is due ** |  |  |
| **Adherence Score**  Total for Mandatory =  Total for Optional =  Overall Total = |  |  |
| **Adherence status (using mandatory variables)**  Total score = 11 …………………………………………………………... **Complete adherence**  Total score = 0-10………………………………………………………….. **Incomplete adherence** | | |
